# Does socio-economic status explain the differentials in malaria parasite prevalence? Evidence from The Gambia

**DOI:** 10.1186/1475-2875-13-449

**Published:** 2014-11-21

**Authors:** Sheriff T Sonko, Malanding Jaiteh, James Jafali, Lamin BS Jarju, Umberto D’Alessandro, Abu Camara, Musu Komma-Bah, Alieu Saho

**Affiliations:** National Malaria Control Programme, Kanifing Institutional Layout, Plot # 17, Kanifing, The Gambia; Center for International Earth Science Information Network (CIESIN), Columbia University, New York, USA; Medical Research Council Laboratories, Fajara, Banjul, The Gambia; Islamic Development Bank, Jeddah, Kingdom of Saudi Arabia; Gambia Bureau of Statistics, Kanifing Institutional Layout, Kanifing, The Gambia

**Keywords:** Malaria, The Gambia, Poorest, Poverty, Socio-economic status

## Abstract

**Background:**

Malaria is commonly associated with poverty. Macro-level estimates show strong links between malaria and poverty, and increasing evidence suggests that the causal link between malaria and poverty runs in both directions. However, micro-level (household and population) analyses on the linkages between malaria and poverty have often produced mixed results.

**Methods:**

The Gambia Malaria Indicator Survey (MIS) 2010/11 was carried out between November 2010 and January 2011. Laboratory-confirmed malaria and wealth quintiles were used to assess the association of socio-economic status and malaria infection in children and the general population. Simple and multiple logistic regressions and survey data analysis procedures, including linearized standard errors to account for cluster sampling and unequal selection probabilities were applied.

**Results:**

Children (six to 59 months) from the second, third, fourth and richest quintiles were significantly less likely to have malaria compared to children from the poorest quintiles. Children (five to 14 years) from the fourth and richest quintiles were also significantly less likely to have malaria compared to those from the poorest quintiles. The malaria burden has shifted from the under-five children (six to 59 months) to children aged five to 14 years. Malaria prevalence was significantly higher in the Central River Region compared to the Upper River Region; and males bear the malaria brunt more than females. Children (six to 59 months) and children (five to 14 years) living in houses with poor walls, floors, roofs and windows were significant associated with higher prevalence of malaria. However, in the general population, only poor wall housing materials were associated with higher prevalence of malaria.

**Conclusions:**

Investments in strategies that address socio-economic disparities and improvements in the quality of housing could, in the long term, significantly reduce the malaria burden in the poorest communities.

## Background

The worldwide malaria burden is currently estimated at about 207 million cases and 627,000 deaths. Sub-Saharan Africa bears the heaviest brunt, with 90% of all deaths, 77% among children under-five. Nevertheless, between 2000 and 2012, malaria mortality rates have decreased by 42% worldwide (by 48% in children under-five) and by 49% in the African Region (by 54% in children under-five) [1].

Malaria is commonly associated with poverty [2–10]. Macro-level estimates show strong links between malaria and poverty. For instance, the malaria burden is highest in the poorest countries, particularly in sub-Saharan Africa, where poverty is widespread and with little economic growth over the past quarter century [5, 6]. The gross national product (GNP) in malaria endemic countries is more than half lower than in non-endemic countries [6]. The poorest 20% of the world’s population contribute to 58% of all malaria deaths [11]. Increasing evidence at macro-level suggests that the causal link between malaria and poverty runs in both directions [3–6].

A review of micro-level (household and population) analyses on the link between malaria and poverty has produced mixed results [12]. Out of nine studies, only two reported a significant association between malaria and poverty [12]. Data from 29 demographic and health surveys (DHS) in 22 countries were used for an aggregate-level regional data analysis for West and Central Africa, and East and Southern Africa, including individual child and country-by-country analysis. No differences were found at the household level in the incidence of fever between the poor and the less poor, though significant differences were found at more aggregate-levels, i.e., country-by-country as opposed to regional-level analysis [13]. In Ghana, social class was not associated with risk of malaria infection [14]. In Tanzania, there was no association between self-reported malaria and socio-economic status (SES) but malaria prevalence was significantly higher among the lower SES individuals [15]. In Nigeria, self-reported malaria or fever was more frequent among the better-off SES and urban dwellers [16] though an earlier study showed a heavier malaria burden among the poor (<US $1/day) compared to the rich [17]. Contrasting results are function of the methodology used to measure malaria and poverty. Studies based on self-reported fever are likely to overestimate the malaria burden while differential reporting of morbidity across socio-economic groups may also influence the results [7, 12].

In 2010/11, The Gambia conducted its first nationwide baseline Malaria Indicator Survey (MIS) in six health regions [18]. The survey covered 4,500 households. Data were collected on long-lasting insecticidal nets (LLINs) coverage and use, malaria case management, intermittent preventive treatment in pregnancy (IPTp), indoor residual spraying (IRS), household assets, general knowledge and practice on malaria prevention and control. In addition, a blood sample to determine the infection status and the haemoglobin level was collected among selected individuals.

In this study, laboratory-confirmed malaria infection and wealth quintiles were used to assess the association between SES including housing quality and malaria infection. The results are reported below.

## Methods

### Management of the MIS 2010/11

The Gambia National Malaria Control Programme (NMCP) in collaboration with The Gambia Bureau of Statistics (GBoS) conducted the MIS 2010/11. A technical working group (TWG), which included representatives from the NMCP, GBoS and other governmental and non-governmental organizations, e.g. National Public Health Laboratories (NPHL), the National Pharmaceutical Services (NPS), the Medical Research Council (MRC), the Catholic Relief Services (CRS), the United Nations Children’s Fund (UNICEF) and the WHO, prepared the protocol and related documents. The GBoS analysed the data.

### Sample design

The sampling frame used for the MIS 2010/11 comprised a complete list of all enumeration areas (EAs) categorized by the eight Local Government Areas (LGAs) and urban–rural residence from the 2003 Population and Housing Census. An EA or cluster is a geographic area with on average 400–700 individuals. According to the 2003 census, there were 2, 475 EAs. For the purpose of sampling, one of the eight LGAs was split into North Bank West Region (NBWR) and North Bank East Region (NBER) to facilitate the recoding of the LGAs into the six health regions at the analysis stage. Thus, for the sample selection, a total of nine LGAs were used.

A two-stage sampling was used to select 4,500 households. The first stage was carried out as a probability proportional to size systematic sampling using the 2003 Population Census Sampling Frame; 225 EAs, 97 in urban and 128 in rural areas were selected. In the second stage, households within the selected EAs were selected randomly from the list of all households within each selected EA prepared by the enumerators. From each EA, twenty households were selected for the interviews. The field coordinators verified the selected households to ensure that there is no selection bias. The enumerators made up to three visits to ensure completeness of the questionnaires, particularly for blood sampling.

The sample size was calculated based on the proportion of children under-five, 49% who slept under an ITN. Each LGA was first allocated with 430 households. This gives 3,870 households. The remaining 630 households were allocated proportional to the 2003 census households to achieve the sample total household count of 4,500. Other parameters considered for the sample size calculation were the average number of children under-five per household (1.1) [19], average household size (8.3 persons) [20], a design effect of 2.0, relative standard error of 10% and a 90% response rate [21].

### Data collection

The MIS 2010/11 started in November 2010 and ended in early January 2011. Two types of questionnaires were used, the household and the women’s questionnaires, administered by 18 teams (two teams per nine LGA; one each for the household data collection and the health component i.e. haemoglobin and parasitaemia testing); each team comprised two supervisors, two enumerators, a nurse, a laboratory assistant and a driver. In addition, there were nine field coordinators whose roles were to ensure adherence to the survey protocol and sampling procedures. Overall, 29 enumerators collected the household data and nine nurses conducted the finger pricks for anaemia and malaria parasitaemia testing.

A blood sample for determining malaria infection and Hb level was collected on all children aged six months to 14 years and women 15–49 years old, including pregnant women, in all selected households. In addition, for every 4^th^ household (general population), all individuals, regardless of age, had a blood sample collected.

A rapid diagnostic test (RDT) was performed to determine malaria infection (SD Bioline Malaria pf/pan), and by thick and thin blood slides. The latter were stained with diluted Giemsa (10%) for 10 minutes. Slides were read independently by two microscopists; slides with discrepant results between microscopy and RDT were read by a third microscopist [18]. Individuals with a positive RDT and with no history of anti-malarial treatment in the previous 14 days were treated with artemether-lumefantrine (AL), the first line treatment in The Gambia. Pregnant women with a positive RDT and with no history of anti-malarial treatment in the previous 14 days received oral quinine if in the first trimester and AL in the second and third trimesters.

Haemoglobin (Hb) was measured with the HaemoCue 201 machine (Ängelholm, Sweden) following the manufacturer’s instructions [18]. Anaemia was defined as haemoglobin (Hb) level <11 g/dl and severe anaemia as Hb <8 g/dl. All subjects with Hb <11 g/dl were treated with ferrous sulphate tablets. Individuals with Hb ≤8 g/dl were treated with iron tablets and referred to the nearest health facility for further management.

Informed consent to participate in the survey was obtained from parents and/or guardians of children aged six months to 12 years, whilst assent was obtained from children aged 13–14 years. For adults, the information sheet about the survey on the questionnaire was read and translated into the local languages to seek their consent to participate in the survey [18].

### Study areas

This study focuses on settlements/villages in the NBWR, CRR and URR thus: “North Bank West Region (NBWR)”, “Central River Region (CRR)” and “Upper River Region (URR)”, the poorest areas of The Gambia where malaria transmission is highest.

(i) The NBWR lies in the northwest of the River Gambia. It is the smallest health region with a total population of 110,970 persons and an annual growth rate of 2.3% between the 2003 and 2013 Censuses. The NBWR has a total land area of 1,054 sq km and a population density of 105 persons per sq km in 2013 [22]. It is the second poorest area with 60% of the population living on less than $1.25 a day [23]. About 15.8% of the population were children under-five, 31% aged five to 14 years and 53.1% aged 15 years and above [22].
(ii) The CRR covers both the north and south banks of the River Gambia. It has a total population of 226,018 persons in 2013, an annual growth rate of 1.9%, and a total land area of 2,894.25 sq km and a population density of 78 persons per sq km. The CRR is home to 12.0% of the overall population of The Gambia [22]. It is the poorest area of the country. The 2010 poverty study showed that 79% and 73.2% of the population, respectively, for the CRR north and CRR south lived on less than $1.25 a day [23]. In 2013, the CRR had 15.7% of its population under-five, 31% aged five to 14 years and 53.3% aged 15 years and above [22].
(iii) The URR lies in the eastern most part of The Gambia. Like the CRR, this region also covers both the north and south banks of the River Gambia. The URR has a total population of 239,916 persons, i.e., 12.7% of the overall population of The Gambia, with an annual growth rate of 2.8% in 2013. The total land area is 2,069.50 sq km and the population density in 2013 was 116 persons per sq km [22]. About 57% of the population live on less than $1.25 a day [23]. Of the total population of the URR in 2013, 15.4% of its children were under-five, 31% aged five to 14 years, and 53.5% aged 15 years and above [22].

### Assets-based wealth quintiles

Data on 27 variables of household possessions and assets, including housing conditions and materials for the construction of walls, roofs, floors and windows were collected. In an earlier analysis of the MIS data, the variables were coded and weights were calculated for use in the construction of indices. The weights were derived using the principal component analysis (PCA) based on Filmer and Pritchett’s [24] method of classifying households into socio-economic groups (SEGs) or quintiles. The results are reassuring as they are consistent with those of the household economic survey [23] cited above, which used income and expenditure methods to measure poverty (Table 1).

**Table 1:** **Percentage distribution of households by wealth quintiles, residence and region, The Gambia MIS 2010/11**

Wealth quintiles							
Residence	Poorest	Second	Middle	Fourth	Richest	Total	Number of households
Urban	0.4	4.5	16.2	35.1	43.8	100	1,908
Rural	34.7	31.7	22.9	8.7	2.1	100	2,538
**Total**	**20**	**20**	**20**	**20**	**20**	**100**	**4,446**
**Health region**							
Western Region	1.1	6.7	16.1	31.1	45.0	100	1,680
North Bank West Region	24.3	32.4	25.7	13.6	4.0	100	448
North Bank East Region	10.7	31.6	27.6	23.0	7.0	100	456
Lower River Region	15.0	41.2	28.9	10.4	4.6	100	454
Central River Region	54.2	23.6	13.2	6.3	2.7	100	937
Upper River Region	29.1	17.0	25.9	20.2	7.9	100	471
**Total**	**20**	**20**	**20**	**20**	**20**	**100**	**4,446**

Source: The Gambia MIS 2010/11 Report, June 2012.

### Production of malaria parasite prevalence maps

Using the MIS 2010/11 dataset, a cross-tabulation of all the 225 selected EAs by the variable malaria result (malresult) by the eight local government areas (LGAs) was performed in stages to obtain malaria parasite prevalence by LGA and EA for the country. The results were transferred to an Excel database; and the district and name of settlements/village(s) were added as identifiers. The malaria parasite prevalence for the country was classified into five categories i.e. no malaria (0%), low (>0% and <5%), moderately-low (≥5% and <10%), moderately-high (≥10% and < 20%), and high (≥20%). The scale cut-offs of the malaria prevalence are standard ones as the different categories are <5%. This could be taken as the threshold for pre-elimination status, 5–9.99, 10–19.9 and then > =20%.

**Figure 1:**
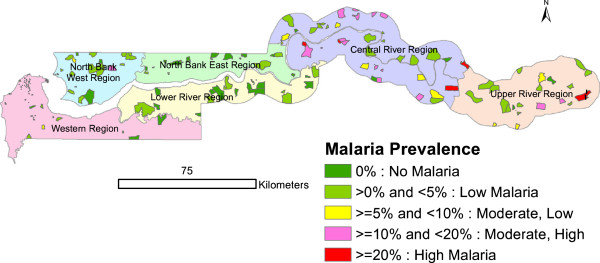
**Malaria parasite prevalence by six health regions, The Gambia MIS 2010/11.**

**Figure 2:**
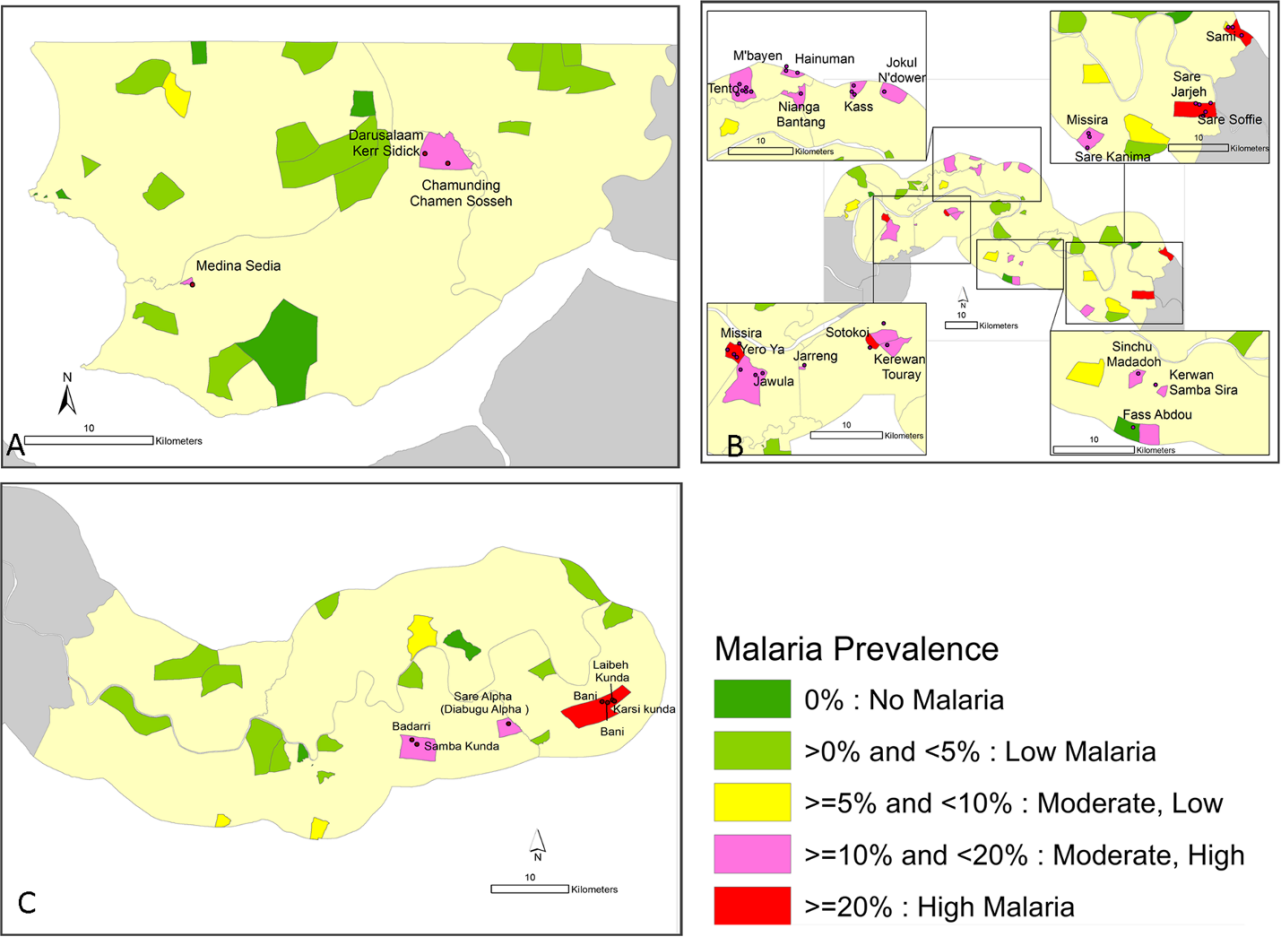
**Malaria parasite prevalence by settlements/villages, North Bank West Region, Central River Region and Upper River Region, The Gambia, MIS 2010/11 (A, B and C).**

These classifications were used to produce a map of The Gambia showing malaria parasite prevalence by six health regions and settlements/villages (Figure 1). In order to have a good view of the settlements/villages with the highest malaria prevalence, three maps were produced separately for the NBWR, CRR and URR with settlement/village names added (Figure 2A, B and C respectively). The unit of analysis for the map is EA or cluster. ArcGIS 10.2.2 Desktop software was used to produce the maps.

### Data management and analysis

A subset of the MIS 2010 dataset (i.e., the low, moderately low and moderately high and high malaria prevalence settlements/villages) was selected for this project. The selected dataset contains 12,274 records. SPSS (version 19) was used for the initial analysis using Pearson’s Chi-square test of significance. All the subsequent data analyses, e.g., production of tables, simple and multiple logistic regressions were performed using STATA version 12.1 (StataCorp LP, College Station, TX, USA). Survey data analysis procedures with Taylor [25] series expansion linearized standard errors to account for cluster sampling and unequal selection probabilities was used. However, the analyses in this report were not weighted. Percentages for categorical variables and means for continuous variables with 95% confidence intervals were estimated.

Simple and multiple logistic regression analyses were applied to assess predictors of malaria prevalence among children six to 59 months, five to 14 years as well as all the age groups combined, including 15 year olds and above in the selected general population (one in four households). The association of SES by wealth quintiles estimated using wealth asset index was assessed. The association of malaria with the type of housing materials used for the construction of walls, floors, roofs, and windows was also assessed. Separate analyses were performed for the children six to 59 months, five to 14 years and the combined age groups, including 15 year olds and above in the selected general population (one in four households). The rationale is to compare with the six to 59 months children and to ascertain if the burden of malaria has shifted from the under-fives to the older children (five to 14 years). The general population was also meant to provide comparable malaria parasite prevalence data with the children under-five (six to 59 months).

All adjusted analyses were controlled for age groups, sex and health regions. Unadjusted and adjusted odds ratios with 95% confidence intervals are presented. Statistical significance was defined as a p-value <0.05. Housing quality was defined based on construction materials used for the floors, walls, roofs, and windows (Table 2).

**Table 2:** **Definitions of housing quality used in the logistic regression model, The Gambia MIS 2010/11**

Building characteristics	Materials used for construction
Floor	Good = (Cement or tiles)
	Poor = (Earth/sand, dung and palm bamboo)
Wall	Good = (Bricks, cement blocks, covered adobe and other)
	Poor = (Cane/palm/trunks, mud/dirt, stone with mud; and mud/krinting*)
Roof	Good = (Sod, metal corrugated iron, cement concrete)
	Poor = (Thatch/palm leaf)
Window	Good = (having a window with at least glasses, screen, curtain, or shutters)
	Poor = (No window at all or window without glasses, screen, curtain, or shutters)

*Krinting is a Wolof word for woven bamboo. Walls are constructed with woven *krinting* and then plastered with mud in the interior and exterior.

### Ethics and approval

The Gambia MIS 2010/11 survey protocol was subjected to rigorous review before approval was granted. The University of The Gambia (UTG) Committee on Research reviewed the first draft of the protocol. The Scientific Coordinating Committee (SCC) of the MRC Unit, The Gambia, reviewed the final protocol and the Joint Gambia Government/MRC Ethics Committee granted approval to conduct the MIS 2010/11.

## Results

Over 18,000 blood slides were collected. The overall malaria prevalence among children aged six to 59 months as determined by microscopy was 4% (5,118). SD Bioline Malaria p/f pan had a sensitivity of 99.0% (95% CIs: 97.5-99.7) and specificity 98.1% (95% CIs: 96.7–99.0), consistent with the manufacturer’s estimates [18].

Malaria parasite prevalence among children was highest in the Central River Region (CRR), 9.9% (1,445) and the Upper River Region (URR), 4.4% (964); and lowest in the North Bank East Region (NBER), 0.5% (527) and the Lower River Region (LRR), 0.8% (481). The Western Region (WR) and the North Bank West Region (NBWR) had malaria parasite prevalence of 2.5% (1,027); and 3.1% (674), respectively [18].

The CRR had the highest percentage of poorest households (54.2%), followed by the URR (29.1%) and the NBWR (24.3%) (Table 1). Prevalence was heterogeneous, with values of 0% or <5% in the WR (Figure 1) although some EAs in the NBWR had higher prevalence (Figure 2A). Most EAs with moderate-high to high prevalence were found in the CRR and the URR i.e. the central and eastern parts of the country (Figure 2B-C).

### Malaria parasite prevalence among children aged six to 59 months and children aged five to 14 years

The overall malaria parasite prevalence among children aged six to 59 months was 10% (1,248), (95% CIs: 7.8-12.7%). Malaria parasite prevalence tended to be higher among children aged six to 11 months: 13.2% (144), (95% CIs: 7.6-21.9%) and among children aged thirty-six to 47 months: 11.9% (301) (95% CIs: 7.9-17.8%). Children from the poorest households were 8.2 times (15.6 *vs* 1.9%) more likely to have had malaria compared to children from the richest households (Table 3).

**Table 3:** **Malaria parasite prevalence among children aged six to 59 months by background characteristics, The Gambia MIS 2010/11**

Background characteristics	N	n	% (95% CI)
**Age (months)**			
6-11	144	19	13.1 (7.5, 21.9)
12-23	273	24	8.7 (5.7, 13.2)
24-35	284	27	9.5 (6.0, 14.5)
36-47	301	36	11.9 (7.8, 17.7)
48-59	246	19	7.7 (4.9, 11.9)
**Total**	**1,248**	**125**	10.0 (7.8, 12.7)
**Sex**			
Male	681	69	10.1 (7.6, 13.3)
Female	567	56	9.8 (7.0, 13.6)
**Total**	**1,248**	**125**	10.0 (7.8, 12.7)
**Health regions**			
Western Region	56	5	8.9 (3.7, 19.8)
North Bank West Region	104	8	7.6 (2.2, 22.9)
Central River Region	714	92	12.8 (9.8, 16.7)
Upper River Region	359	19	5.2 (2.4, 10.9)
**Total**	1,233*	**124**	10.0 (7.8, 12.8)
**Wealth Index**			
Poorest	500	78	15.6 (12.3, 19.4)
Second	224	16	7.1 (4.0, 12.3)
Middle	260	22	8.4 (4.9, 14.1)
Fourth	146	6	4.1 (2.0, 8.2)
Richest	103	2	1.9 (0.7, 5.3)
**Total**	**1,248**	**125**	10.0 (7.8, 12.7)

*Total excludes missing cases.

The overall malaria parasite prevalence among children aged five to 14 years was 11% (1,987), (95% CIs: 8.4-14.3%). Children from the poorest households were 2.6 times (15.2 *vs* 5.8%) more likely to have had malaria compared to children from the richest households (Table 4).

**Table 4:** **Malaria parasite prevalence among children aged five to 14 years by background characteristics, The Gambia MIS 2010/11**

Background characteristics	N	n	% (95% CI)
**Age (years)**			
5-9	1,094	115	10.5 (7.5, 14.5)
10-14	893	104	11.6 (8.7,15.3)
**Total**	**1,987**	**219**	11.0 (8.4, 14.3)
**Sex**			
Male	1,003	117	11.6 (8.6, 15.5)
Female	984	102	10.3 (7.6, 13.8)
**Total**	**1,987**	**219**	11.0 (8.4, 14.3)
**Health regions**			
Western Region	74	6	8.1 (6.1, 10.6)
North Bank West Region	175	15	8.5 (4.6, 15.2)
Central River Region	1,054	131	12.4 (9.3, 16.2)
Upper River Region	656	65	9.9 (4.9, 18.9)
**Total**	1,959*	**217**	11.0 (8.4, 14.4)
**Wealth index**			
Poorest	717	109	15.2 (11.5, 19.7)
Second	358	34	9.5 (5.1, 16.8)
Middle	416	50	12.0 (8.3, 16.9)
Fourth	314	15	4.7 (2.4, 9.0)
Richest	154	9	5.8 (3.7, 8.9)
**Total**	**1,987**	**219**	**11.0 (8.4, 14.3)**

*Total excludes missing cases.

### Malaria parasite prevalence in the general population

In the general population, the overall malaria parasite prevalence was 8.7% (2,306), (95% CIs: 7.4-10.2%). Parasite prevalence was highest among children aged five to 14 years, 10.5% (693), (95% CIs: 8.6-12.9%). Children from the poorest households were 3.7 times (11.1 *vs* 3.0%) more likely to have had malaria compared to children from the richest households (Table 5).

**Table 5:** **Malaria parasite prevalence in the general population by age and background characteristics, The Gambia MIS 2010/11**

Background characteristics	N	n	% (95% CI)
**Age**			
6-59 months	394	32	8.1 (5.6, 11.4)
5-14 years	693	73	10.5 (8.5, 12.9)
15+	1,219	96	7.8 (6.2, 9.9)
**Total**	**2,306**	**201**	8.7 (7.4, 10.2)
**Sex**			
Male	1,065	108	10.1 (8.2, 12.4)
Female	1,241	93	7.4 (5.9, 9.4)
**Total**	**2,306**	**201**	8.7 (7.4, 10.2)
**Health regions**			
Western Region	114	8	7.0 (3.5, 13.4)
North Bank West Region	195	9	4.6 (1.9, 10.7)
Central River Region	1,180	128	10.8 (8.8, 13.2)
Upper River Region	765	48	6.2 (4.6, 8.3)
**Total**	2,254*	**193**	8.5 (7.2, 10.1)
**Wealth index**			
Poorest	863	96	11.1 (8.7, 14.1)
Second	484	38	7.8 (5.2, 11.5)
Middle	456	40	8.7 (6.5, 11.7)
Fourth	286	14	4.9 (3.2, 7.3)
Richest	165	5	3.0 (0.9, 9.1)
**Total**	**2,306**	**201**	**8.7 (7.4, 10.2)**

*Total excludes missing cases.

### Multivariate analysis

Among children six to 59 months old, there were no significant associations between malaria and age (p = 0.438), health region (p = 0.133) and sex (p = 0.621). After adjusting for age, sex and health region, children from the second (OR = 0.42; 95% CI: 0.25; 0.73, p = 0.002), third (OR = 0.52; 95% CI: 0.29; 0.95, p = 0.03), fourth (OR = 0.27; 95% CI: 0.12; 0.61, p = 0.002) and richest (OR = 0.12; 95% CI: 0.05; 0.33, p < 0.001) quintiles were significantly less likely to have malaria compared to children from the poorest quintiles. Having poor walls (OR = 1.87; 95% CI: 1.12; 3.11, p = 0.01), floors (OR = 2.0; 95% CI: 1.4; 3.0, p = 0.001), roofs (OR = 2.2; 95% CI: 1.4; 3.3, p < 0.001), and windows (OR = 1.9; 95% CI: 1.2; 3.0, p = 0.01) was significantly associated with higher prevalence of malaria even after adjusting for age, sex and health regions (Table 6).

**Table 6:** **Factors associated with malaria parasite prevalence among children aged six to 59 months, The Gambia MIS, 2010/11**

Variables		Unadjusted		Adjusted	
	N	OR (95% CI)	P value	OR (95% CI)	P value
**Age group (months)**		1.0 (0.9;1.050)	0.6		
06-11	144	1		1	0.4
12-23	273	0.6 (0.3;1.2)	0.1	0.7 (0.3;1.2)	0.2
24-35	284	0.7 (0.3;1.6)	0.3	0.7 (0.3;1.7)	0.5
36-47	301	0.9 (0.4;1.9)	0.7	1.0 (0.5;2.2)	0.9
48-59	246	0.6 (0.2;1.3)	0.1	0.6 (0.2;1.4)	0.2
**Health regions**					
Western Region	56	1		1	0.1
North Bank West Region	104	0.8 (0.2;4.1)	0.8	0.5 (0.1;2.4)	0.4
Central River Region	714	1.5 (0.6;4.0)	0.4	0.6 (0.3;1.4)	0.3
Upper River Region	359	0.6 (0.2;1.9)	0.3	0.4 (0.1;1.0)	0.06
**Sex**					
Male	681	1		1	0.6
Female	567	0.9 (0.6;1.4)	0.8	0.9 (0.6;1.3)	
**Wealth quintiles**					
Poorest	500	1		1	
Second	224	0.4 (0.2;0.8)	0.009	0.4 (0.2;0.7)	0.002
Third	260	0.5 (0.2;0.9)	0.03	0.5 (0.3;0.9)	0.03
Fourth	146	0.2 (0.1;0.5)	0.001	0.3 (0.1;0.6)	0.002
Richest	103	0.1 (0.04;0.3)	<0.001	0.1 (0.04;0.3)	<0.001
**Wall type**					
Good	451	1		1	
Poor	797	2.3 (1.3;4.1)	0.005	1.8 (1.1;3.1)	0.01
**Floor type**					
Good	644	1		1	
Poor	604	2.5 (1.5;3.9)	<0.001	2.0 (1.4;3.0)	0.001
**Roof type**					
Good	799	1		1	
Poor	449	2.4 (1.6;3.7)	<0.001	2.2 (1.4;3.3)	<0.001
**Window type**					
Good	730	1		1	
Poor	513	2.0 (1.3;3.0)	0.001	1.8 (1.1;3.0)	0.01

Among children aged five to 14 years old, there were no significant associations between malaria and age (p = 0.4), health region (p = 0.2) and sex (p = 0.4). Children from the fourth (OR = 0.2; 95% CI: 0.1; 0.5, p < 0.001) and richest (OR = 0.3; 95% CI: 0.1; 0.6, p = 0.003) quintiles were significantly less likely be infected compared to those from the poorest quintiles. However, there were no significant differences in malaria prevalence between children from the poorest quintiles and those from the second (p = 0.1) and third (p = 0.1) quintiles. Having poor walls (OR = 1.8; 95% CI: 1.1; 3.0, p = 0.01), floors (OR = 1.5; 95% CI: 1.0; 2.2, p = 0.03), roofs (OR = 1.6; 95% CI: 1.1; 2.3, p = 0.01), and windows (OR = 1.7; 95% CI: 1.3; 2.3, p < 0.001) were significantly associated with higher prevalence of malaria, even after adjusting for age, sex and health regions (Table 7).

**Table 7:** **Factors associated with malaria parasite prevalence among children aged five to 14 years, The Gambia MIS, 2010/11**

Variables		Unadjusted		Adjusted	
	N	OR (95% CI)	P value	OR (95% CI)	P value
**Age (years)**		1.0 (0.9;1.0)	0.8		
**Age group (years)**					
5-9	1094	1		1	0.4
10-14	893	1.1 (0.8;1.6)	0.5	1.1 (0.8;1.6)	
**Health regions**					
Western Region	74	1		1	0.2
North Bank West Region	175	1.0 (0.5;2.2)	0.9	0.6 (0.3;1.3)	0.2
Central River Region	1054	1.6 (1.0;2.5)	0.03	0.8 (0.4;1.4)	0.4
Upper River Region	656	1.2 (0.5;2.8)	0.6	1.0 (0.5;1.9)	0.9
**Sex**					
Male	1003	1		1	0.4
Female	984	0.9 (0.6;1.2)	0.4	0.9 (0.6;1.2)	
**Wealth quintiles**					
Poorest	717	1		1	
Second	358	0.6 (0.3;1.1)	0.1	0.6 (0.3;1.2)	0.1
Third	416	0.8 (0.5;1.2)	0.2	0.7 (0.5;1.1)	0.1
Fourth	314	0.3 (0.1;0.6)	0.001	0.2 (0.1;0.5)	0.000
Richest	154	0.3 (0.2;0.6)	<0.001	0.3 (0.1;0.6)	0.003
**Wall type**					
Good	796	1		1	
Poor	1191	1.7 (1.1;2.7)	0.011	1.8 (1.1;3.0)	0.02
**Floor type**					
Good	1094	1		1	
Poor	893	1.6 (0.9;2.5)	0.07	1.5 (1.0;2.2)	0.03
**Roof type**					
Good	1343	1		1	
Poor	643	1.7 (1.2;2.5)	0.006	1.6 (1.1;2.3)	0.015
**Window type**					
Good	1228	1		1	
Poor	750	1.8 (1.4;2.3)	<0.001	1.7 (1.3;2.3)	<0.001

In the general population, i.e. all household members in every 4^th^ household, malaria prevalence was significantly lower in the fourth (OR = 0.4; 95% CI: 0.2; 0.8, p = 0.01) and richest (OR = 0.2; 95% CI: 0.08; 0.8, p = 0.02) quintiles compared to the poorest quintiles. Having poor wall housing materials was associated with higher prevalence of malaria (OR = 1.6; 95% CI: 1.1; 2.3, p = 0.01). However, floors, roofs and windows were not significantly associated with malaria infection (Table 8).

**Table 8:** **Factors associated with malaria parasite prevalence among the general population, The Gambia MIS, 2010/11**

Variables		Unadjusted		Adjusted	
	N	OR (95% CI)	P value	OR (95% CI)	P value
**Age (years)**		1.0 (0.1;1.0)	0.1		
**Age group (years)**					
<5	394	1		1	
5-14	693	1.3 (0.8;2.1)	0.2	1.4 (0.9;2.1)	0.1
15+	1219	1.0 (0.6;1.4)	0.8	1.0 (0.7;1.4)	0.8
**Health Region**					
Western Region	114	0.6 (0.3;1.3)	0.2	1.4 (0.6;3.6)	0.5
North Bank West Region	195	0.4 (0.2;1.0)	0.05	0.4 (0.2;1.0)	0.05
Central River Region	1180	1		1	0.02
Upper River Region	765	0.5 (0.4;0.8)	0.003	0.74 (0.5;1.00)	0.05
**Sex**					
Male	1065	1		1	0.09
Female	1241	0.7 (0.5;1.0)	0.04	0.8 (0.5;1.0)	
**Wealth quintiles**					
Poorest	863	1		1	0.05
Second	484	0.7 (0.4;1.1)	0.2	0.8 (0.4;1.3)	0.3
Third	456	0.8 (0.5;1.2)	0.2	0.8 (0.5;1.2)	0.3
Fourth	286	0.4 (0.2;0.7)	0.001	0.4 (0.2;0.8)	0.01
Richest	165	0.2 (0.08;0.8)	0.02	0.2 (0.07;0.8)	0.02
**Wall type**					
Good	850	1		1	
Poor	1456	1.8 (1.3;2.6)	0.002	1.6 (1.1;2.3)	0.01
**Floor type**					
Good	1245	1		1	
Poor	1061	1.6 (1.1;2.4)	0.01	1.3 (0.8;2.2)	0.2
**Roof type**					
Good	1546	1		1	
Poor	757	1.8 (1.2;2.7)	0.004	1.5 (1.0;2.4)	0.07
**Window type**					
Good	1309	1		1	
Poor	991	1.3 (0.9;1.9)	0.2	1.2 (0.8;1.7)	0.4

## Discussion

Malaria prevalence was strongly associated with SES, even after adjusting for age, sex and health region, regardless of the age group. These findings are consistent with previous studies carried out in sub-Saharan Africa in which other measures of SES such as occupation, housing type, including the uptake of interventions, e.g., ITNs and expenditure on chemoprophylaxis were associated with malaria infection [12, 26–31].

The recent findings by Tusting *et al.*[9], is also consistent with this study. However, these findings differ from Somi *et al.*[15], who found no association between malaria and SES, and Somi *et al*. [32], who found negative associations between malaria and SES running in both directions. In addition, the findings of this study are contrary to a study [16] in southeast Nigeria based on self-reported malaria or fever, which found more malaria among better-off SES and urban dwellers compared to the poor.

Age, health region and sex were not significantly associated with malaria prevalence across sub-population groups. However, malaria prevalence was significantly higher in the CRR compared to the URR, with males being more at risk than females. It is interesting noticing that the CRR is the poorest in The Gambia followed by the URR [23, 33].

The high malaria infection in the CRR and the URR (i.e. eastern parts of the country) can be explained by ecological factors such as fresh water, floodplains, swamp and upland rice fields, higher temperatures and lower humidity compared to the WR (western half of the country). Studies have shown that swamp rice fields are important habitat for mosquito larvae [34, 35].

The malaria burden has also shifted from under-five children (six to 59 months) to children aged five to 14 years; OR = 1.4 (95% CI: 0.9; 2.1), p = 0.168, see Table 8). The reasons for the shift in malaria burden from the under-fives to the older children aged five to 14 years are unclear. One hypothesis is that The Gambia NMCP had initially over focused (i.e., activities and information, education and communication (IEC) messages) on the under-fives (0–59 months), particularly for ITN coverage. However, the finding is consistent with a study in northeast Tanzania, which showed that children aged five to 13 years were now at a higher risk of malaria compared to under-five children. The odds of having malaria were also higher for males compared to females in northeast Tanzania and Ethiopia [28, 36].

Historically, malaria elimination in the USA, Italy, Greece, and Spain was achieved through socio-economic development and intensive anti-malarial interventions, such as improved housing. Doors and windows were screened to reduce contacts between mosquitoes and people coupled with combined environmental management, e.g., draining of swampland to eliminate breeding grounds of certain vector mosquitoes and IRS using DDT [6, 9]. For example, in The Gambia, a randomized controlled trial of full screening of doors and windows and closing of eaves showed a 50% decline in the risk of anaemia-related malaria among children [37]. This randomized controlled trial has proved that improvements in housing quality can greatly reduce malaria transmission and incidence.

Interventions aiming at improving the living conditions of populations in endemic communities can have a major impact on the malaria burden [4]. This is also supported by the finding that housing construction materials, e.g., type of wall, floor, roof, and window, are strongly associated with the risk of infection in most age groups, though in the general population this was true only for poor wall housing materials. The association with type of housing and risk of malaria has already been described in other countries. In Mozambique [38], Eritrea [39, 40] and the Lao People’s Democratic Republic, living in houses with grass roof was associated with higher odds of malaria infection [41]. Similarly, houses constructed with bamboo walls, poorly fitted windows and doors, and often inhabited by people from low SES were associated with mosquito house entry [41]. Living in houses with no windows or screens is likely to increase individual contact with the mosquito vector [42]. Mud used as wall material was associated with high prevalence of malaria [43].

However, the study has several limitations. The analysis did not include women aged 15–49 years who were pregnant at the time of the survey due to the paucity of the data (only 148 women). Nonetheless, compared to the other subpopulations in this study, pregnant women had the lowest malaria parasite prevalence of 6.8% (95% CIs: 2.7-10.9%). Also, interventions such as ITN/LLIN ownership and use and IRS were not included due to the paucity of data.

Over the last decade, The Gambia has made significant progress in reducing the malaria burden. All-cause under-five mortality declined from 141 deaths per 1,000 in 2002 to 131 deaths per 1,000 in 2007 [19] and further declined significantly to 109 deaths per 1,000 in 2012 [44]. A new Malaria Strategic Plan and Policy for the period 2014–2020 [45, 46] with a vision of a ‘malaria-free Gambia by 2020’ has been prepared. Nevertheless, malaria transmission is still ongoing, despite the achieved high coverage of preventive and curative interventions. There may be the need of additional interventions targeting the human reservoir of infection and aiming at interrupting transmission.

The findings of this study pose major challenges for malaria prevention and control interventions in The Gambia. First, there is the need to address the unacceptably high malaria burden among the poorest communities in the CRR, URR and NBWR. Studies have shown that malaria infections and mosquito vectors are not homogenously distributed within population groups and areas. Thus, it is argued that countries address the heterogeneity in malaria transmission long before they enter the pre-elimination phase [43, 47, 48]. Second, there is the need to consolidate the gains made as The Gambia moves towards the malaria pre-elimination phase. Increased funding is critical in achieving these objectives. Currently, the country is the only one in the sub-region where malaria interventions are funded from a single source, the Global Fund for AIDS, Tuberculosis and Malaria (GFATM). Funding is therefore a major challenge, which threatens the prospects for sustained malaria decline.

## Conclusions

The evidence suggests that malaria infection was strongly associated with low SES. Children from the poorest quintiles, living in poor housing conditions were more at risk of having malaria infection. While intensifying the existing malaria interventions may reduce malaria in the short term, investments in strategies that address socio-economic disparities and improvements in the quality of housing could, in the long term, significantly reduce the malaria burden in the poorest communities.

## Abbreviations

AL: Artemether-lumefantrine
CRR: Central River Region
EAs: Enumeration areas
GBoS: Gambia Bureau of Statistics
Hb: Haemoglobin
IPTp: Intermittent preventive therapy during pregnancy
IRS: Indoor residual spraying
ITNs: Insecticide-treated nets
LGAs: Local government areas
LLINs: Long-lasting insecticide-treated nets
LRR: Lower River Region
MIS: Malaria Indicator Survey
MoHSW: Ministry of Health and Social Welfare
MRC: Medical Research Council
NBER: North Bank East Region
NBWR: North Bank West Region
NMCP: National Malaria Control Programme
OR: Odds ratio
RBM: Roll-Back Malaria
RDTs: Rapid diagnostic tests
SES: Socio-economic status
SPSS: Statistical Package for the Social Sciences
TWG: Technical working group
URR: Upper River Region
WHO: World Health Organization
WR: Western Region.
